# Recombinant Production, SpeciesSpecific Activity at the TRPA1 Channel, and Significance of the N-Terminal Residue of ProTx-I Toxin from Thrixopelma Pruriens Tarantula Venom

**DOI:** 10.32607/actanaturae.27590

**Published:** 2025

**Authors:** M. A. Shulepko, M. Zhang, E. A. Zhivov, D. S. Kulbatskii, A. S. Paramonov, Yu. Che, A. V. Kuznetsov, A. V. Popov, M. P. Kirpichnikov, Z. O. Shenkarev, E. N. Lyukmanova

**Affiliations:** Faculty of Biology, Shenzhen MSU-BIT University, Shenzhen, Guangdong Province, 518172 China; Shemyakin–Ovchinnikov Institute of Bioorganic Chemistry, Moscow, 117997 Russia; Moscow Center for Advanced Studies, Moscow, 123592 Russia; Kurchatov Medical Primatology Center of National Research Center “Kurchatov Institute”, Krasnodarskiy kray, Sochi, 354376 Russia; Interdisciplinary Scientific and Educational School “Molecular Technologies of the Living Systems and Synthetic Biology”, Faculty of Biology, Lomonosov Moscow State University, Moscow, 119234 Russia

**Keywords:** cystine knot, TRPA1 channel, gating modifier toxin, bacterial production, disulfide-rich proteins

## Abstract

The ProTx-I toxin from Thrixopelma pruriens tarantula venom inhibits voltage-gated sodium (NaV), potassium, and calcium channels, as well as the chemosensitive TRPA1 ion channel, affecting the activating processes of these channels. Due to its action at the NaV1.7, NaV1.8, and TRPA1 channels involved in pain perception and propagation, ProTx-I may be used as a model for the development of next-generation analgesics. ProTx-I consists of 35 amino acid residues, with three disulfide bonds forming an inhibitor cystine knot (ICK) motif, which challenges its heterologous production. An efficient ProTx-I production system is necessary to study, at the molecular level, the mechanism by which the toxin acts. In this study, we tested several approaches for bacterial production of disulfide-containing toxins. Cytoplasmic expression of ProTx-I fused with either thioredoxin or glutathione-S-transferase failed to yield a correctly folded toxin. However, the natively folded ProTx-I was successfully obtained by “direct” expression in the form of cytoplasmic inclusion bodies, followed by renaturation, as well as by secretion into the periplasmic space via fusion with maltose-binding protein. The activity of the recombinant ProTx-I was studied by electrophysiology in X. laevis oocytes expressing rat and human TRPA1 channels. The toxin proved to be more active on the rat channel than on the human channel (IC_50_ = 250 ± 85 and 840 ± 190 nM, respectively). The presence of an additional N-terminal methionine residue in the toxin obtained through “direct” expression significantly attenuated its activity.

## INTRODUCTION

Spider venoms are a rich source of polypeptide toxins that act on various membrane receptors and ion channels [1, 2, 3]. Many spider toxins belong to the knottin family that includes small (20–50 aa) β-structural peptides containing a conserved inhibitory cystine knot (ICK) motif [4] formed by three disulfides: C1–C4, C2–C5, and C3–C6. This spatial structure is responsible for the high physicochemical and proteolytic stability of knottins, making the ICK motif a promising basis for the design of new peptide drugs [5].

Spider knottins include membrane-active toxins that affect the activation or inactivation of sodium (NaV), potassium, and calcium voltage-gated channels (so-called gating modifier toxins) [6]. The ProTx-I toxin (Protoxin-I or β/ω-theraphotoxin-Tp1a, 35 aa) is a membrane-active knottin of the Peruvian green velvet tarantula Thrixopelma pruriens. ProTx-I effectively inhibits a number of voltage-gated channels [7], as well as the chemosensitive TRPA1 channel [8]. Among the ProTx-I targets, NaV1.7, NaV1.8, and TRPA1 channels are promising therapeutic targets for the treatment of pain and neurological inflammatory syndromes [9, 10, 11]. Studying the mechanism of ProTx-I action on these channels may yield valuable insight that could help in the development of new analgesics and other biomedical drugs.

The first step that is required in order to study the mechanism of ProTx-I action and, eventually, design new variants of this knottin is to develop an efficient production system. Traditionally, small polypeptide toxins, including spider knottins, are produced using methods of peptide synthesis followed by refolding to form the correct system of disulfide bonds [12]. In addition, recombinant knottins are produced in Pichia pastoris cells [12, 13, 14] and Escherichia coli cells [15, 16]. However, during cytoplasmic production, these proteins accumulate as insoluble inclusion bodies [17, 18]. In E. coli cells, disulfide-rich toxins are produced through (1) “direct” expression followed by isolation of the peptide from inclusion bodies and its refolding; (2) fusion with proteins that promote the formation of disulfide bonds and increase the level of production, e.g., thioredoxin A (TRX) or glutathione S-transferase (GST); and (3) secretion of recombinant peptides into the E. coli periplasmic space where formation of disulfide bonds occurs [17, 19].

We compared these approaches in bacterial ProTx-I production and, for the first time, produced a correctly folded recombinant toxin and characterized its activity at human and rat TRPA1 channels. The obtained data demonstrate a significant species-specificity of the inhibitory action of ProTx-I, as well as the influence of the N-terminal sequence of the toxin on its activity. The developed bacterial production system opens new opportunities for the production of mutant and isotope-labeled ProTx-I variants for further structural and functional studies.

## EXPERIMENTAL

**Design of expression vectors**

**Fig. 1:**
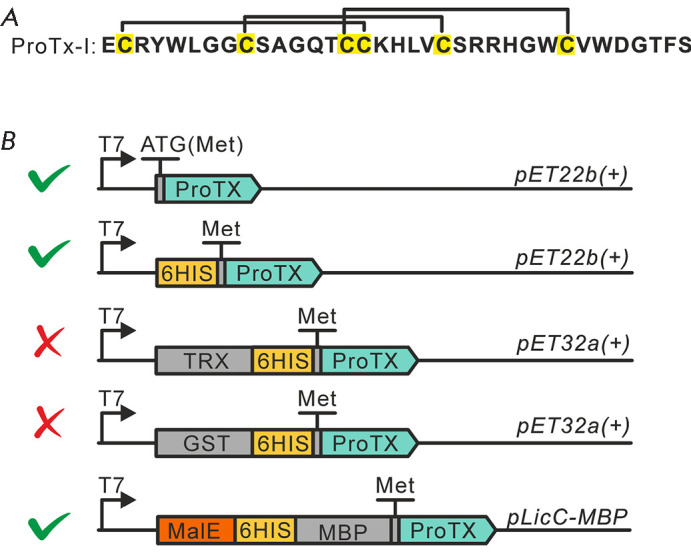
Design of expression vectors for ProTx-I production in E. coli cells. (A) The amino acid sequence of the ProTx-I toxin. Cysteine residues are shown in yellow; disulfide bonds are indicated with solid lines. (B) Schematic representation of expression vectors. From top to bottom: vectors for “direct” expression of Met-ProTx-I and 6His-Met-ProTx-I; vectors for the cytoplasmic production of ProTx-I with the partners TRX and GST; the vector for ProTx-I secretion as a fusion protein with MBP. Check marks indicate the vectors successfully used for the production of correctly folded ProTx-I

The ProTx-I gene was constructed based on the amino acid sequence P83480 from the UniProt database. The nucleotide sequence of the gene was optimized in accordance with the codon usage frequency in E. coli. Vectors for the cytoplasmic expression of the TRX-ProTx-I and GST-ProTx-I fusion proteins were prepared by cloning the ProTx-I gene into the pET-32a(+) (Novagene, USA) and pET-32a(+)/GST vectors at the KpnI/BamHI and BamHI/HindIII sites, respectively. The pET-32a(+)/GST plasmid was produced prior by replacing the TRX gene sequence in the pET-32a(+) plasmid with the GST gene sequence. The vector for bacterial secretion of the MBP-ProTx-I fusion protein was generated by cloning the ProTx-I gene into the pLicC-MBP-APETx2 plasmid (Addgene, #72668) at the KpnI and SacI sites [20]. The vector for direct expression of MetProTx-I was prepared by cloning the ProTx-I gene into the pET-22b(+) vector (Novagene) at the NdeI and BamHI sites. In this case, the N-terminus of the ProTx-I molecule contained an additional methionine residue encoded by the ATG start codon. To produce 6His-Met-ProTx-I, an additional sequence encoding the 6His-tag and a linker sequence containing a methionine residue were inserted at the 5’-end of the ProTx-I gene. This gene was then cloned into the pET-22b(+) vector at the NdeI and BamHI sites. Schematic representations of the constructs used in the study are shown in Fig. 1.

**Bacterial production of the TRX-ProTx-I, GST-ProTx-I, and MBP-ProTx-I fusion proteins**

To produce the TRX-ProTx-I and GST-ProTx-I fusion proteins, E. coli BL21(DE3) and SHuffle T7 Express (NEB) strains were transformed with the pET-32a(+)/TRX-ProTx-I and pET-32a(+)/ GST-ProTx-I vectors, respectively. MBP-ProTx-I was produced in the E. coli Rosetta-gami (DE3) strain. Cells were grown in a TB medium (12 g of bactotryptone, 24 g of yeast extract, 4 mL of glycerol, 2.3 g of KH_2_ PO4 , 12.5 g of K2 HPO4 per 1 L of medium, pH 7.4) containing 100 μg/mL ampicillin (Sigma, USA) to OD_600_ ~ 0.6. Expression was induced by adding 0.1 mM isopropyl-β-D-1-thiogalactopyranoside (IPTG, Sigma). The cells were cultivated at 20°C for 16 h for GST-ProTx-I or at 13°C for 72 h for MBP-ProTx-I and TRX-ProTx-I.

**Bacterial production of Met-ProTx-I and 6His-Met-ProTx-I**

Met-ProTx-I and 6His-Met-ProTx-I were produced in the E. coli BL21(DE3) strain transformed with the pET-22b(+)/Met-ProTx-I or pET-22b(+)/6His-Met-ProTx-I vector. To produce Met-ProTx-I, cells were grown in a TB medium at 37°C to OD_600_ of ~0.6 and expression was induced by adding 0.2 mM IPTG. To produce 6His-Met-ProTx-I, cells were grown in a SB medium (32 g of bactotryptone, 20 g of yeast extract, 5 g of NaCl, pH 7.4) at 37°C to OD_600_ of ~6.0 and expression was induced with 1 mM IPTG. After induction, cell cultivation was continued at 37°C for 18 h.

**Isolation and purification of the TRX-ProTx-I, GST-ProTx-I, and MBP-ProTx-I fusion proteins**

Cells were collected by centrifugation at 10,000 g and 4°C for 20 min. The cell pellet was resuspended in buffer A (20 mM Tris-HCl, 300 mM NaCl, pH 8.0) in the presence of 1 mM phenylmethylsulfonyl fluoride (PMSF, Sigma). The cells were disrupted by ultrasound (Branson Digital Sonifier, USA) at an output power of 500 W and 4°C for 6 min. The suspension was centrifuged at 30,000 g at 4°C for 30 min. Fusion proteins were purified by metal-chelate affinity chromatography on a Ni-Sepharose FastFlow resin (Cytiva, USA) pre-equilibrated in buffer A. Recombinant proteins were eluted by imidazole (Macklin, China) gradient (20–500 mM).

**Isolation and purification of reduced Met-ProTx-I and 6His-Met-ProTx-I**

Isolation of Met-ProTx-I from cytoplasmic inclusion bodies and its purification under denaturing conditions were performed according to the protocols reported elsewhere [19]. After chromatography, Met-ProTx-I was reduced with a 500-fold molar excess of dithiothreitol (DTT, Sigma). Cytoplasmic inclusion bodies containing 6His-Met-ProTx-I were solubilized in denaturing buffer (20 mM Tris-HCl, 300 mM NaCl, 10 mM β-mercaptoethanol, 8 M urea, pH 8.0) for 3 h, centrifuged, and the supernatant was applied on a Ni-Sepharose FastFlow chromatography resin equilibrated with denaturing buffer. 6His-Met-ProTx-I was eluted by imidazole gradient (20–500 mM). Before BrCN hydrolysis (Sigma), chromatographic fractions of 6His-Met-ProTx-I were supplemented with a 500-fold molar excess of DTT.

**Hydrolysis of recombinant proteins with BrCN**

The recombinant proteins 6His-Met-ProTx-I, TRX-ProTx-I, GST-ProTx-I, and MBP-ProTx-I at a concentration of 4 mg/mL were hydrolyzed by adding 0.3 M HCl and a 50-fold molar excess (relative to methionine residues) of BrCN. The reaction proceeded in the dark at room temperature overnight. BrCN was then removed by evaporation using a Centrivap (Labconco, USA) equipped with a cryogenic trap.

**Renaturation of Met-ProTx-I and ProTx-I**

Renaturation of Met-ProTx-I and ProTx-I (produced by BrCN hydrolysis of 6His-Met-ProTx-I) was initiated by transferring the recombinant proteins to the refolding buffer (0.1 M Tris-HCl, 2 M urea, 1.5 mM GSH, and 0.15 mM GSSG, pH 7.5) using gel filtration on NAP-25 chromatography columns (Cytiva). The final concentration of recombinant toxins in the refolding buffer was 0.02 mg/mL.

**Purification and analysis of recombinant ProTx-I variants by HPLC**

HPLC analysis of recombinant ProTx-I variants was performed on a Jupiter C4 column (A300, 4.6 × 250 mm, Phenomenex) using the Vanquish Core and Ultimate 3000 chromatographs (ThermoFisher, USA). Toxins were eluted by an acetonitrile gradient containing 0.1% trifluoroacetic acid (TFA) at a flow rate of 1 mL/min. The resulting toxin samples were lyophilized.

**Mass spectrometry**

**Fig. 2:**
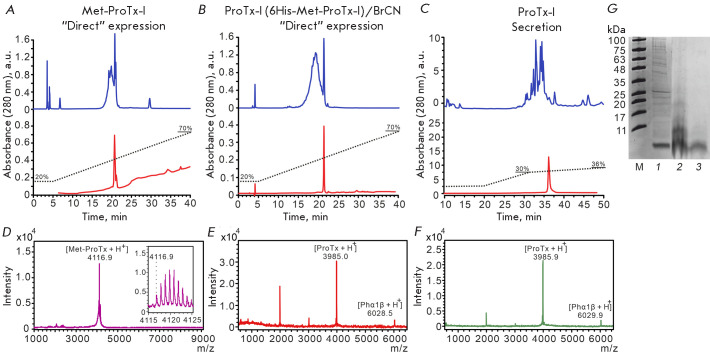
Purification and characterization of recombinant ProTx-I variants. (A–C) Representative HPLC chromatograms of the purification (upper panel) and analysis (lower panel) of recombinant ProTx-I variants. (A) Refolded Met-ProTx-I. (B) ProTx-I produced by “direct” expression of 6His-Met-ProTx-I, followed by BrCN hydrolysis and refolding. (C) ProTx-I produced by bacterial secretion of MBP-ProTx-I, followed by BrCN hydrolysis. (D–F) MALDI-MS spectra of recombinant ProTx-I variants shown in (A–C), respectively. The Phα1β toxin was added to the samples shown in (E and F). [ProTx-I+2H^+^] and [Phα1β+2H^+^] doubly charged ions are also observed in (E and F). (G) Electrophoretic analysis of the ProTx-I samples produced by “direct” expression: M, marker (BioSharp BL712A); 1, Met-ProTx-I before refolding; 2, ProTx-I after hydrolysis of 6His-Met-ProTx-I with BrCN; 3, ProTx-I as in lane 2 after refolding

The Met-ProTx-I sample was analyzed using a Rapiflex MALDI-TOF/TOF spectrometer (Bruker, Germany) in the reflection positive ion mode. The resulting m/z value (4116.9 Da, Fig. 2D) was close to an expected monoisotopic [Met-ProTx-I + H^+^] mass of 4116.7 Da for a toxin molecule with closed disulfide bonds.

Analysis of the ProTx-I variants produced by the hydrolysis of 6His-Met-ProTx-I (Fig. 2E) or bacterial secretion (Fig. 2F) was performed using the Ultraflex MALDI-TOF/TOF spectrometer (Bruker, Germany). Trypsin autolysis products were used for spectrometer calibration. The molecular masses of ProTx-I (3985.0 and 3985.9 Da) achieved in both cases corresponded to the calculated monoisotopic ProTx-I mass (3985.7 Da, [ProTx-I + H^+^]) within the measurement error. In both cases, the samples additionally contained the Phα1β toxin (calculated mass 6029.5 Da [Phα1β + H^+^]) to verify calibration and its measured mass was 6028.5 and 6029.9 Da, respectively.

**NMR spectroscopy**

NMR spectra were measured in an aqueous solution (5% D2 O, pH 4.5, 30°C) using an AVANCE-800 NMR spectrometer (Bruker) with an operating proton frequency of 800 MHz. A commercial toxin sample obtained by chemical synthesis (Smartox Biotechnology Inc., France) was used as a positive control for the correct spatial structure.

**Electrophysiological experiments**

Currents through human and rat TRPA1 [21] were recorded in X. laevis oocytes expressing these channels. Oocyte isolation, mRNA injection, and experimental recordings have been described previously [22]. All solutions were prepared on the day of the experiment using calcium-free ND-96 containing 96 mM NaCl, 2 mM KCl, 1 mM MgCl_2_ , and 10 mM HEPES at pH 7.4. Currents were stimulated by the application 100 μM AITC (Sigma-Aldrich). The solution was manually added to the perfusion chamber, and the currents were recorded using voltage ramps as reported in [22]. For each oocyte, we sequentially recorded three responses to AITC application, as well as the subsequent leakage current in the presence of a specific TRPA1 inhibitor: HC030031 (Sigma-Aldrich). The first response amplitude was used to normalize the data obtained on different oocytes. To induce the second response, AITC was applied together with ProTx-I or HC030031. The amplitude of this response was measured, normalized, averaged across different oocytes, and used to plot dose-response curves. Dose-response curves were approximated by the Hill equation:

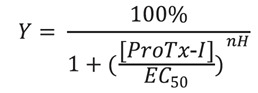



where nH is the Hill coefficient.

Statistical data processing was performed using GraphPad Prism 9.0. To compare current amplitudes at specific toxin concentrations, either a two-tailed Student’s t-test or one-way ANOVA and Dunnett’s multiple comparison test was used. Dose-response curve parameters were compared using the F-test.

## RESULTS AND DISCUSSION

**Production of ProTx-I as a fusion protein with TRX and GST**

Five different approaches were tested to produce recombinant ProTx-I. Toxin was produced as a soluble fusion protein with TRX, GST, and maltose-binding protein (MBP), and in the form of cytoplasmic inclusion bodies with and without six N-terminal histidine residues – 6His-tag (Fig. 1). The efficiency of recombinant production of spider toxins fused with TRX and GST was demonstrated previously [23, 24, 25, 26], and the efficiency of production as cytoplasmic inclusion bodies, followed by refolding, has been demonstrated by us for a number of disulfide-rich proteins, including snake venom toxins and human proteins from the Ly6/uPAR family [27].

**Fig. 3:**
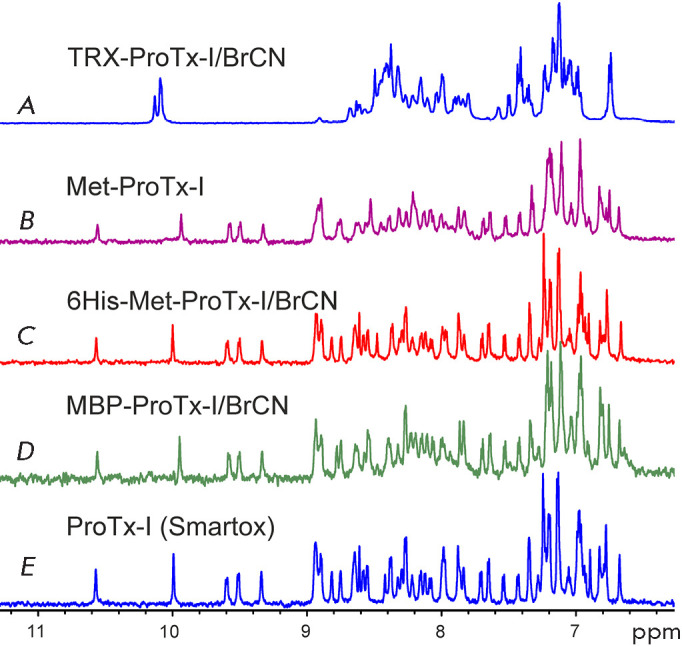
1D 1 H NMR spectra of recombinant ProTx-I variants (pH 4.5, 30°C). (A) The spectrum of an incorrectly folded ProTx-I produced as a TRX-ProTx-I fusion protein, followed by BrCN hydrolysis. (B) The spectrum of a refolded Met-ProTx-I. (C) The spectrum of ProTx-I obtained by “direct” expression of the 6His-Met-ProTx-I, followed by BrCN hydrolysis and refolding. (D) The spectrum of ProTx-I produced by secretion of an MBP-ProTx-I fusion protein followed by BrCN hydrolysis. (E) The spectrum of commercial, synthetic ProTx-I

Cultivation of BL21(DE3) cells transformed with the pET-32a/ProTx-I plasmid at 37°C resulted in the production of an insoluble TRX-ProTx-I fusion protein, whereas reducing the cell culture temperature to 13°C resulted in the production of a soluble protein with a yield of 20 mg/L of bacterial culture. Since the ProTx-I molecule lacks methionine residues, we used BrCN to hydrolyze the fusion protein [28] at an additional methionine residue introduced upstream of the first ProTx-I residue. MALDI analysis of the purified ProTx-I sample confirmed the expected molecular mass of the toxin with closed disulfide bonds. However, comparison of the 1 H NMR spectra of the recombinant toxin and commercial ProTx-I revealed absence of the correct spatial structure for recombinant ProTx-I (Fig. 3A,E). Employing GST as a protein partner also failed to yield a correctly folded ProTx-I. These results underscore the need for an analysis of the spatial structure of recombinant toxins before any further analysis. Production of a soluble fusion protein and verification of its molecular mass do not guarantee proper protein folding and the formation of correct disulfide bonds.

**Production of Met-ProTx-I in inclusion bodies**

By “direct” expression, the ProTx-I gene contains the ATG start codon at the 5’-end, which is necessary for translation initiation. Therefore, a final recombinant protein produced as cytoplasmic inclusion bodies contains an additional N-terminal methionine residue (Fig. 1B). To purify Met-ProTx-I, we used a previously developed protocol that involved cytoplasmic inclusion body solubilization to obtain a denatured toxin with cysteine residues chemically modified to S-sulfonate, followed by ion-exchange chromatography on a DEAP-spheronite-OH resin [27]. The yield of denatured Met-ProTx-I at this stage was 6 mg/L of bacterial culture. The purified Met-ProTx-I was treated with DTT to remove S-sulfonate groups from cysteine residues, then DTT was removed by gel filtration, and the toxin was transferred to the renaturation buffer. The toxin refolding protocol was based on a protocol previously reported for chemically synthesized ProTx-I [29] and was similar to the protocols we used for the refolding of other disulfide-rich proteins [27]. Refolded Met-ProTx-I, purified by HPLC (Fig. 2A), had the native spatial structure (Fig. 3B), but the efficiency of toxin refolding using this approach was extremely low. The final yield of the refolded toxin was only ~0.05 mg/L of bacterial culture.

**Production of ProTx-I with the native N-terminal sequence**

The low efficiency of Met-ProTx-I refolding may be due to insufficient purity of the sample before the refolding procedure. To overcome this problem, a 6His-tag was introduced into the N-terminal sequence of ProTx-I. A similar approach was previously used for recombinant production of other spider toxins [30]. Additional residues in the N-terminal sequence of toxins can affect their structure and activity [31]. Therefore, to produce a toxin with the native N-terminal sequence, we introduced an additional methionine residue after the 6His-tag for subsequent BrCN hydrolysis (Fig. 1B). The yield of 6His-Met-ProTx-I after purification by metal-chelate chromatography was ~13 mg/L of bacterial culture. Thus, the introduction of the 6His-tag into the N-terminal sequence not only increased the purity of the toxin preparation before renaturation (Fig. 2G) but also elevated the level of toxin production, which is consistent with our earlier observation that the N-terminal sequence can affect the yield of recombinant proteins [32]. The denatured 6HisMet-ProTx-I was hydrolyzed with BrCN, and the refolding was performed similarly to that for the Met-ProTx-I. The final yield of the refolded ProTx-I with the native N-terminal sequence after HPLC (Fig. 2B) was 0.3 mg/L of bacterial culture. Thus, the introduction of the 6His-tag into the ProTx-I molecule not only increased the production level of the toxin but also yielded a folded peptide with the native sequence, which was confirmed by NMR spectroscopy (Fig. 3C).

**Secretion of ProTx-I**

An alternative approach for the production of proteins with correctly formed disulfide bonds in E. coli cells is secretion into the periplasmic space [20]. To enhance toxin production, we used the peptide fused with MBP. To secrete the fusion protein into the periplasmic space, we introduced the MalE signal peptide into the N-terminal sequence of MBP [33] (Fig. 1B). Also, a methionine residue was inserted before the toxin sequence to cleave ProTx-I from MBP. For this step, we used E. coli Rosetta-gami™ (Origami™ derivative), which had proven itself to be effective in the production of disulfide-rich proteins, including animal toxins [34, 35]. To increase the yield of soluble protein, we lowered the cell culture temperature after induction to 13°C, which slowed the rate of protein synthesis and promoted the correct formation of disulfide bonds [36]. The yield of the MBP-ProTx-I protein after purification from a total cell lysate using metal chelate affinity chromatography was 75 mg/L of bacterial culture. The MBP-ProTx-I protein was then hydrolyzed with BrCN, and ProTx-I with the native N-terminal sequence was purified by HPLC (Fig. 2C). The final yield of the secreted, correctly folded ProTx-I (Fig. 3D) was ~0.15 mg/L of bacterial culture. Minor differences in the positions of individual signals in the NMR spectra of recombinant and commercial toxins (Fig. 3D,E) are explained by pH variations (within 0.1 units) in the samples.

**The N-terminal sequence of ProTx-I influences toxin-TRPA1 interaction**

**Fig. 4:**
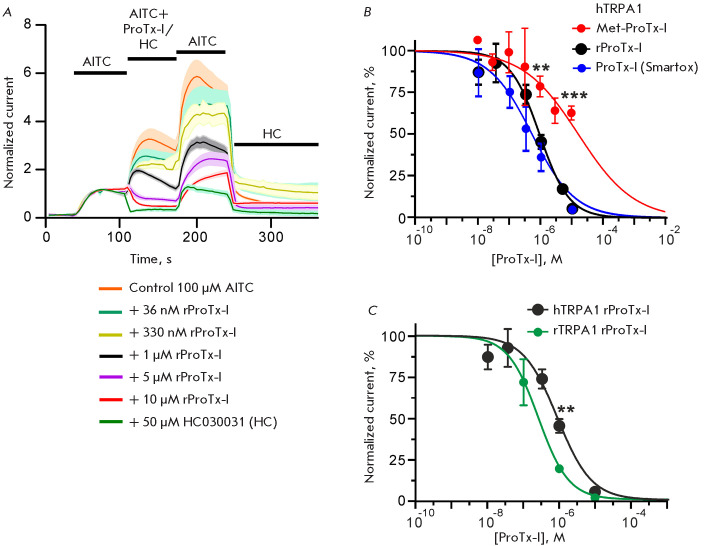
Effect of ProTx-I variants on AITC-induced outward currents through TRPA1 in X. laevis oocytes. (A) Average normalized current traces for the human TRPA1 channel in the absence and presence of the selective antagonist HC030031 or ProTx-I. Data are presented as mean ± SEM (lines and shaded areas, respectively, n = 3–6 oocytes). Compound application periods are shown by solid black bars above the traces. (B) Dose-response curves for the inhibition of human TRPA1 by recombinant rProTx-I, Met-ProTx-I, and commercial synthetic ProTx-I (Smartox). **(p < 0.01) and ***(p < 0.001) indicate a significant difference in the current amplitudes between Met-ProTx-I and other variants according to the ANOVA/Dunnett criterion. The difference in IC_50_ values for the corresponding curves approximated by the Hill equation (Table 1) is statistically significant with p < 0.0001 (F-test). (C) Dose-response curves for recombinant rProTx-I at human (hTRPA1) and rat (rTRPA1) ion channels. The difference in IC_50_ values for these curves is statistically significant with p = 0.006 (F-test). **(p < 0.01) indicates a significant difference in the amplitude of currents at rat and human channels according to the two-sided t-test. Data in B and C (mean ± SEM, n = 3–6 oocytes) are normalized to the response recorded without ProTx-I (100%)

Activity of the chemically synthesized ProTx-I was previously demonstrated in HEK293 cells expressing human and mouse TRPA1 receptors [8]. In the present study, we compared the functional activity of Met-ProTx-I and ProTx-I, with the latter being produced by hydrolysis of 6His-Met-ProTx-I, at the human TRPA1 channel expressed in X. laevis oocytes. Consistent with [8], we found that 10 μM of recombinant ProTx-I almost completely inhibited the current through the TRPA1 channel induced by 100 μM of the covalent agonist allyl isothiocyanate (AITC) (Fig. 4A). This effect was similar to that of 50 μM HC0300301, a selective TRPA1 antagonist (Fig. 4A).

Comparison of the dose-response curves of recombinant and commercial ProTx-I confirmed their similarity (Fig. 4B, Table 1). The curve parameters (IC_50_ and Hill coefficient) were not statistically different. However, analysis of Met-ProTx-I revealed a dramatic, statistically significant decline in the activity of this toxin variant. For example, recombinant and synthetic ProTx-I at a concentration of 10 μM inhibited currents to ~5%, whereas Met-ProTx-I inhibited currents only to ~60%, with the IC_50_ value increased by an order of magnitude (Fig. 4B, Table 1). Thus, the N-terminal amino acid sequence of the toxin is critically important for its interaction with the receptor. It is noteworthy that previous data on the ProTx-I active site had not included the N-terminal residues [8].

**Table 1:** The Hill equation parameters for the inhibition curve analysis

Receptor/toxin	IC50, µM	Hill coefficient
hTRPA1/Met-ProTx-I	8.9 ± 7.0	= 1.0^*^
hTRPA1/ProTx-I (Smartox)	0.41 ± 0.16	0.68 ± 0.24
hTRPA1/rProTx-I	0.84 ± 0.19	0.91 ± 0.18
rTRPA1/rProTx-I	0.25 ± 0.07	1.03 ± 0.26

^*^The Hill coefficient was set equal to 1.0 to analyze the dose-response curve for this toxin variant.

**ProTx-I inhibits rat TRPA1 more efficiently than the human channel**

Comparison of recombinant ProTx-I activity at rat and human TRPA1 channels revealed higher activity of the toxin at the rat receptor (IC_50_ ~ 250 and 840 nM, respectively; the difference in IC_50_ was statistically significant, Fig. 4B, Table 1). It is noteworthy that a previous comparative study of ProTx-I at human and mouse TRPA1, conversely, revealed higher activity at the human channel [8]. The difference in the toxin’s action on human, rat, and mouse receptors may be explained by significant differences in the amino acid sequences of the extracellular S1-S2 and S3-S4 loops of these TRPA1 channels, the primary site of toxin interaction [8]. For example, the conserved residue Glu754 (numbering is given for the human channel) is replaced by Gly in the mouse channel, and Glu825 in the human channel is replaced by Asp and Asn in the rat and mouse channels, respectively. There are also other point differences. As a result, two negatively charged residues in the toxin-binding site of the mouse channel are replaced by neutral residues, which probably attenuates the binding of the positively charged toxin molecule (charge +2).

## CONCLUSION

A system for the recombinant production of the ProTx-I toxin in the folded state was developed for the first time. We showed that ProTx-I exhibits different activities relative to human and rat TRPA1 channels, and that modification of the N-terminal sequence of ProTx-I may lead to toxin inactivation.

## Abbreviations

AITC: allyl isothiocyanate
GST: glutathione S-transferase
ICK: inhibitory cystine knot
MBP: maltose-binding protein
NaV: voltage-gated sodium channel
TRX: thioredoxin
IPTG: isopropyl β-D-1-thiogalactopyranoside

## References

[1] Kuhn-Nentwig L., Stöcklin R., Nentwig W. (2011). Venom Composition and Strategies in Spiders. Is Everything Possible?. Adv Insect Physiol..

[2] Peigneur S., de Lima ME., Tytgat J. (2018). Phoneutria nigriventer venom: A pharmacological treasure.. Toxicon..

[3] Lyukmanova EN., Shenkarev ZO. (2024). Toxins from Animal Venom − A Rich Source of Active Compounds with High Pharmacological Potential.. Toxins (Basel)..

[4] Cardoso FC., Lewis RJ. (2019). Structure-Function and Therapeutic Potential of Spider Venom-Derived Cysteine Knot Peptides Targeting Sodium Channels.. Front Pharmacol..

[5] Kintzing JR., Cochran JR. (2016). Engineered knottin peptides as diagnostics, therapeutics, and drug delivery vehicles.. Curr Opin Chem Biol..

[6] Milescu M., Bosmans F., Lee S., Alabi AA., Kim JI., Swartz KJ. (2009). Interactions between lipids and voltage sensor paddles detected with tarantula toxins.. Nat Struct Mol Biol..

[7] Middleton RE., Warren VA., Kraus RL. (2002). Two tarantula peptides inhibit activation of multiple sodium channels.. Biochemistry..

[8] Gui J., Liu B., Cao G. (2014). A Tarantula-Venom Peptide Antagonizes the TRPA1 Nociceptor Ion Channel by Binding to the S1–S4 Gating Domain.. Curr Biol..

[9] Maatuf Y., Geron M., Priel A. (2019). The Role of Toxins in the Pursuit for Novel Analgesics.. Toxins (Basel)..

[10] Souza Monteiro de Araujo D., Nassini R., Geppetti P., De Logu F. (2020). TRPA1 as a therapeutic target for nociceptive pain.. Expert Opin Ther Targets..

[11] Dormer A., Narayanan M., Schentag J. (2023). A Review of the Therapeutic Targeting of SCN9A and Nav1.7 for Pain Relief in Current Human Clinical Trials.. J Pain Res..

[12] Moore SJ., Cochran JR. (2012). Engineering knottins as novel binding agents.. Methods Enzymol..

[13] Fitches EC., Pyati P., King GF., Gatehouse JA. (2012). Fusion to snowdrop lectin magnifies the oral activity of insecticidal ω-Hexatoxin-Hv1a peptide by enabling its delivery to the central nervous system.. PLoS One..

[14] Yang S., Pyati P., Fitches E., Gatehouse JA. (2014). A recombinant fusion protein containing a spider toxin specific for the insect voltage-gated sodium ion channel shows oral toxicity towards insects of different orders.. Insect Biochem Mol Biol..

[15] Monfared N., Ahadiyat A., Fathipour Y., Mianroodi RA. (2022). Evaluation of recombinant toxin JFTX-23, an oral-effective anti-insect peptide from the spider Selenocosmia jiafu venom gland proteome.. Toxicon..

[16] Matsubara FH., Meissner GO., Herzig V. (2017). Insecticidal activity of a recombinant knottin peptide from Loxosceles intermedia venom and recognition of these peptides as a conserved family in the genus.. Insect Mol Biol..

[17] Costa S., Almeida A., Castro A., Domingues L. (2014). Fusion tags for protein solubility, purification and immunogenicity in Escherichia coli: the novel Fh8 system.. Front Microbiol..

[18] Berlec A., Strukelj B. (2013). Current state and recent advances in biopharmaceutical production in Escherichia coli, yeasts and mammalian cells.. J Ind Microbiol Biotechnol..

[19] Klint JK., Senff S., Saez NJ. (2013). Production of recombinant disulfide-rich venom peptides for structural and functional analysis via expression in the periplasm of E. coli.. PLoS One..

[20] Anangi R., Rash LD., Mobli M., King GF. (2012). Functional Expression in Escherichia coli of the Disulfide-Rich Sea Anemone Peptide APETx2, a Potent Blocker of Acid-Sensing Ion Channel 3.. Mar Drugs..

[21] Logashina YA., Solstad RG., Mineev KS. (2017). New Disulfide-Stabilized Fold Provides Sea Anemone Peptide to Exhibit Both Antimicrobial and TRPA1 Potentiating Properties.. Toxins (Basel)..

[22] Lyukmanova EN., Mironov PA., Kulbatskii DS. (2023). Recombinant Production, NMR Solution Structure, and Membrane Interaction of the Phα1β Toxin, a TRPA1 Modulator from the Brazilian Armed Spider Phoneutria nigriventer.. Toxins (Basel)..

[23] Berkut AA., Peigneur S., Myshkin MY. (2015). Structure of Membrane-active Toxin from Crab Spider Heriaeus melloteei Suggests Parallel Evolution of Sodium Channel Gating Modifiers in Araneomorphae and Mygalomorphae.. J Biol Chem..

[24] Shlyapnikov YM., Andreev YA., Kozlov SA., Vassilevski AA., Grishin EV. (2008). Bacterial production of latarcin 2a, a potent antimicrobial peptide from spider venom.. Protein Expr Purif..

[25] Paiva ALB., Matavel A., Peigneur S. (2016). Differential effects of the recombinant toxin PnTx4(5-5) from the spider Phoneutria nigriventer on mammalian and insect sodium channels.. Biochimie..

[26] Zhang H., Huang PF., Meng E. (2015). An efficient strategy for heterologous expression and purification of active peptide hainantoxin-IV.. PLoS One..

[27] Shulepko MA., Lyukmanova EN., Shenkarev ZO. (2017). Towards universal approach for bacterial production of three-finger Ly6/uPAR proteins: Case study of cytotoxin I from cobra N. oxiana.. Protein Expr Purif..

[28] Andreev YA., Kozlov SA., Vassilevski AA., Grishin EV. (2010). Cyanogen bromide cleavage of proteins in salt and buffer solutions.. Anal Biochem..

[29] Rupasinghe DB., Herzig V., Vetter I. (2020). Mutational analysis of ProTx-I and the novel venom peptide Pe1b provide insight into residues responsible for selective inhibition of the analgesic drug target NaV1.7.. Biochem Pharmacol..

[30] Vásquez-Escobar J., Benjumea-Gutiérrez DM., Lopera C. (2023). Heterologous Expression of an Insecticidal Peptide Obtained from the Transcriptome of the Colombian Spider Phoneutria depilate.. Toxins (Basel)..

[31] Dubovskii PV., Dubinnyi MA., Konshina AG. (2017). Structural and Dynamic “Portraits” of Recombinant and Native Cytotoxin I from Naja oxiana: How Close Are They?. Biochemistry..

[32] Lyukmanova EN., Shenkarev ZO., Khabibullina NF. (2012). N-terminal fusion tags for effective production of g-protein-coupled receptors in bacterial cell-free systems.. Acta Naturae..

[33] Saez NJ., Cristofori-Armstrong B., Anangi R., King GF. A. (2017). Strategy for Production of Correctly Folded Disulfide-Rich Peptides in the Periplasm of E. coli.. Methods Mol Biol..

[34] Li J., Zhang H., Liu J., Xu K. (2006). Novel genes encoding six kinds of three-finger toxins in Ophiophagus hannah (king cobra) and function characterization of two recombinant long-chain neurotoxins.. Biochem J..

[35] Clement H., Flores V., De la Rosa G., Zamudio F., Alagon A., Corzo G. (2016). Heterologous expression, protein folding and antibody recognition of a neurotoxin from the Mexican coral snake Micrurus laticorallis.. J Venom Anim Toxins Incl Trop Dis..

[36] Lyukmanova EN., Shulga AA., Arsenieva DA. (2004). A Large-Scale Expression in Escherichia coli of Neurotoxin II from Naja oxiana Fused with Thioredoxin.. Russ J Bioorg Chem..

